# Single energy CT-based mass density and relative stopping power estimation for proton therapy using deep learning method

**DOI:** 10.3389/fonc.2023.1278180

**Published:** 2023-11-23

**Authors:** Yuan Gao, Chih-Wei Chang, Justin Roper, Marian Axente, Yang Lei, Shaoyan Pan, Jeffrey D. Bradley, Jun Zhou, Tian Liu, Xiaofeng Yang

**Affiliations:** ^1^ Department of Radiation Oncology and Winship Cancer Institute, Emory University, Atlanta, GA, United States; ^2^ Department of Biomedical Informatics, Emory University, Atlanta, GA, United States; ^3^ Perelman School of Medicine, University of Pennsylvania, Philadelphia, PA, United States; ^4^ Department of Radiation Oncology, Icahn School of Medicine at Mount Sinai, New York, NY, United States

**Keywords:** deep learning, CT, relative stopping power, mass density, proton therapy

## Abstract

**Background:**

The number of patients undergoing proton therapy has increased in recent years. Current treatment planning systems (TPS) calculate dose maps using three-dimensional (3D) maps of relative stopping power (RSP) and mass density. The patient-specific maps of RSP and mass density were obtained by translating the CT number (HU) acquired using single-energy computed tomography (SECT) with appropriate conversions and coefficients. The proton dose calculation uncertainty of this approach is 2.5%-3.5% plus 1 mm margin. SECT is the major clinical modality for proton therapy treatment planning. It would be intriguing to enhance proton dose calculation accuracy using a deep learning (DL) approach centered on SECT.

**Objectives:**

The purpose of this work is to develop a deep learning method to generate mass density and relative stopping power (RSP) maps based on clinical single-energy CT (SECT) data for proton dose calculation in proton therapy treatment.

**Methods:**

Artificial neural networks (ANN), fully convolutional neural networks (FCNN), and residual neural networks (ResNet) were used to learn the correlation between voxel-specific mass density, RSP, and SECT CT number (HU). A stoichiometric calibration method based on SECT data and an empirical model based on dual-energy CT (DECT) images were chosen as reference models to evaluate the performance of deep learning neural networks. SECT images of a CIRS 062M electron density phantom were used as the training dataset for deep learning models. CIRS anthropomorphic M701 and M702 phantoms were used to test the performance of deep learning models.

**Results:**

For M701, the mean absolute percentage errors (MAPE) of the mass density map by FCNN are 0.39%, 0.92%, 0.68%, 0.92%, and 1.57% on the brain, spinal cord, soft tissue, bone, and lung, respectively, whereas with the SECT stoichiometric method, they are 0.99%, 2.34%, 1.87%, 2.90%, and 12.96%. For RSP maps, the MAPE of FCNN on M701 are 0.85%, 2.32%, 0.75%, 1.22%, and 1.25%, whereas with the SECT reference model, they are 0.95%, 2.61%, 2.08%, 7.74%, and 8.62%.

**Conclusion:**

The results show that deep learning neural networks have the potential to generate accurate voxel-specific material property information, which can be used to improve the accuracy of proton dose calculation.

**Advances in knowledge:**

Deep learning-based frameworks are proposed to estimate material mass density and RSP from SECT with improved accuracy compared with conventional methods.

## 1 Introduction

The number of patients receiving proton therapy treatment is rising each year. Current proton treatment planning systems (TPS) incorporate relevant proton energy deposition physics into the process, determining the patient irradiation pattern. All patients treated with radiotherapy (including protons) are simulated using computed tomography (CT) typically acquired using a single energy scanning protocol. This data is utilized for geometrical implementation of the therapy and also to characterize the patient from the point of view of the probability of charged particle interactions. Therefore, proton dose calculation accuracy is dependent on the capability of TPS to characterize patient tissues based on CT imaging (1). This is done by correlating the CT number of tissue substitute phantoms with known material composition with mass density or relative stopping power (RSP) via the stoichiometric calibration method (2). The accuracy of this approach relies on the difference between patient tissue chemical composition and the tissue substitute database used in the calibration (3–5). Since single-energy computed tomography (SECT) can’t differentiate changes in CT number as a result of differences in either mass density or material chemical composition (6), the error in RSP calculation can become significant. The accuracy of mass density estimation dominates the uncertainty of RSP (7). Furthermore, tissue heterogeneity, CT image noise, and artifacts can also contribute to the RSP calculation error. The direct consequence of uncertainties associated with material characterization (mass density) from SECT data is a loss of accuracy in the prediction of energy deposition relative to the depth of proton interaction, also called proton range uncertainty. To mitigate range uncertainty, TPS have options to allow for the addition of margins in the proton beam range, the standard being 2.5%-3.5% of the energy-dependent range plus an additional 1mm-1.5mm (8).

To further increase the proton therapy therapeutic ratio advantage, many efforts have been made to decrease the proton range uncertainty. One approach is introducing Monte-Carlo dose calculation algorithms to proton TPS (8–12), which can reduce the margin down to 2.4% plus 1.2 mm (8, 13). Another proposed avenue was to use dual-energy CT (DECT) to build calibration curves between CT number and mass density (14). The methodology allows for the acquisition of CT scans with different X-ray spectra, which in turn can be used to determine relative electron density and mean excitation energy (15). Furthermore, DECT virtual monochromatic image reconstruction techniques can reduce beam hardening artifacts and noise. Numerous algorithms for DECT-based RSP estimation have been developed, and the reported results indicate that the errors in RSP estimates can be reduced to 1% (15–17).

As a robust implementation platform for DECT applications in the area of RSP mapping, machine learning (ML) algorithms have also been applied. Su et al. reported their approach for generating parametric maps using ML, which produced accurate effective atomic numbers, relative electron density, mean excitation energy, and RSP from DECT data (18), and they concluded that artificial neural network (ANN) outperformed other reported ML methods. Building on the potential advantage of increased network depth and compositionality (19, 20), deep learning (DL) is an extension of machine learning, which consists of massive multilayered networks or artificial neurons that can discover useful features in CT images (21). DL methods were successfully applied to improve mass density and RSP mapping from DECT datasets (22).

Despite all DECT-based ML and DL applications for improving proton dose calculation, SECT is still the current standard in clinical CT simulators for proton therapy. Therefore, an accurate and efficient method to reduce range uncertainty based on SECT images would benefit existing proton radiotherapy clinics and workflows. DL networks have high degrees of freedom of modeling, therefore offering the opportunity to improve the accuracy of SECT-based RSP and mass density modeling, as has been shown in DECT-based studies. In this study, we investigate the feasibility of using DL models to correlate SECT-based CT numbers to voxel-specific mass density and RSP using two types of electron density phantoms.

## 2 Materials and methods

### 2.1 Phantom SECT data sets

**Figure 1:**
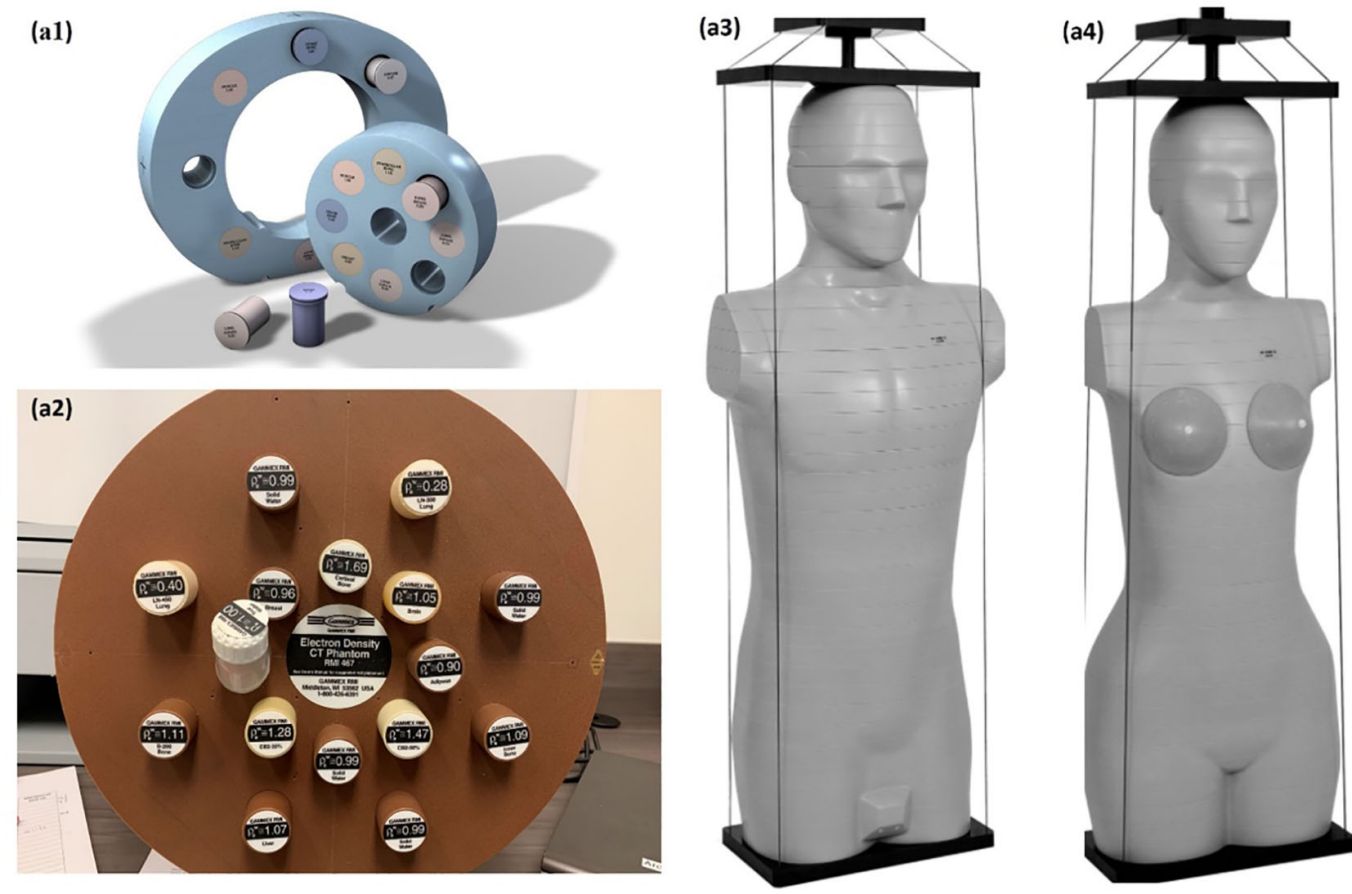
Experiment set up, CIRS 062M electron density phantom (Computerized Imaging Reference Systems) **(A1)**, Gammex 468 electron density phantom **(A2)** CIRS M701 **(A3)**, and CIRS M702 (Computerized Imaging Reference Systems) **(A4)** were scanning with Siemens SOMATOM Definition Edge scanner.

A CIRS 062M electron density phantom (Computerized Imaging Reference Systems, Inc., Norfolk, VA, USA) was used to generate the DL training dataset, while Gammex 467-1009 electron density phantom, CIRS ATOM M701 (male) and M702 (female) anthropomorphic phantoms were chosen to generate the DL networks prediction datasets (**Figure 1**). **Table 1** details the mass density and RSP value for the phantoms used. All mass density information was provided by the manufacturer except the bone insert, for which measurements were utilized to produce a reference value (11). All RSP values were calculated using a previously reported method (22). Phantoms were imaged using a Siemens SOMATOM Definition Edge CT scanner and clinical 120 kVp single energy beam acquisition protocols. The electron density phantom was scanned using a standard head-and-neck protocol. A pelvis protocol was used for the Gammex electron density phantom, while the M701 and M702 phantoms were scanned using three different protocols: head-and-neck (HN), thorax, and pelvis protocols. The manufacturer-reported CTDI_vol_ is reported in **Table 2**, as well as the reconstructed image resolution. All the reconstructed SECT images have a reconstructed field-of-view diameter of 500 mm and a slice thickness of 0.5 mm.

**Table 1:** Phantom insert data: mass densities and RSP.

	Tissue surrogate	*ρ* (g/cm^3^)	RSP
CIRS 062M	Lung (Inhale)	0.203	0.202
	Lung (Exhale)	0.494	0.492
	Adipose	0.965	0.977
	Breast Tissue	0.996	1.003
	Muscle	1.059	1.059
	Liver	1.072	1.070
	Bone 200 mg/cc	1.157	1.116
	Bone 800 mg/cc	1.520	1.404
	Bone 1250 mg/cc	1.830	1.647
Gammex 467-1009	LN450 Lung	0.450	0.448
	Breast	0.980	0.969
	Brain	1.050	1.061
	Liver	1.090	1.090
	B200 Bone Mineral	1.150	1.099
	CB2-50%CaCO_3_ Bone	1.560	1.422
	SB3 Cortical Bone	1.820	1.614
CIRS M701 & M702	Lung	0.202	0.201
	Breast	0.991	0.982
	Soft Tissue	1.055	1.041
	Spinal Cord	1.065	1.035
	Brain	1.069	1.049
	Bone	1.517^1^	1.410

**^1^** The bone mass density was measured.

**Table 2:** CTDI_vol_ and voxel grid spacing information at each site with specified standard acquisition protocol.

	CTDI_vol_ (mGy)/Voxels		
	HN	Thorax	Pelvis
CIRS 062m	23.3/512×512×495		
Gammex 467			23.3/512×512×30
CIRS Atom M701	23.6/512×512×605	23.3/512×512×609	23.3/512×512×471
CIRS Atom M702	23.6/512×512×713	23.3/512×512×881	23.3/512×512×809

### 2.2 Deep learning models

**Figure 2:**
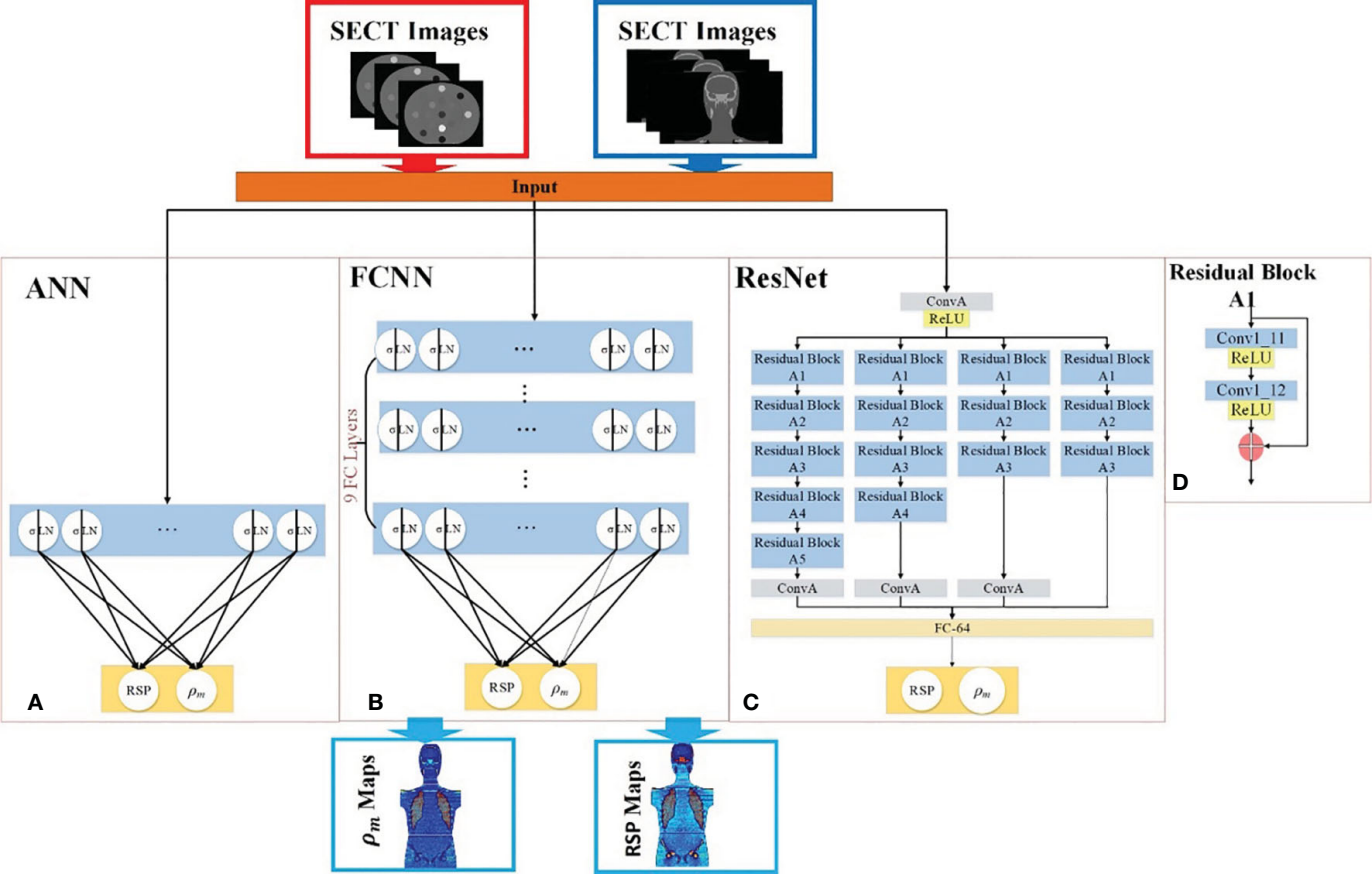
Deep learning framework for mass density and RSP map prediction based on SECT images. The red arrows illustrate the training workflows, while the blue arrows depict the prediction workflow. The framework includes **(A)** ANN, **(B)** FCNN, **(C)** ResNet, and **(D)** the residual block of ResNet. During the training phase (indicated by red arrows), the phantom SECT images are input into the three deep learning networks: ANN, FCNN, and ResNet. For the prediction phase, the M701 and M702 phantoms are fed into the trained deep learning networks to predict mass density and RSP.

Three supervised DL models were implemented to demonstrate the capability of artificial intelligence (AI) to improve proton range calculation using SECT (21, 23–25). **Figure 2A** shows the artificial neural network (ANN) workflow, and **Figures 2B, C** show the fully convolutional neural network (FCNN) and residual neural network (ResNet). 120 kVp spectra SECT images are used as DL input. The same DL models are utilized to estimate both the mass density and RSP relative to the input SECT CT number values. The DL models were supervised by a loss function, defined as the difference between the true value and predicted value at each voxel. All the DL models were implemented in PyTorch (26). Su et al. reported that ANN with 30 hidden units outperforms traditional ML models in generating quantitative parametric maps based on DECT images (18). Their ANN design was adopted for SECT parametric mapping in this study, with 30 hidden hyperbolic-tangent (tanh) layers and error backpropagation (see **Figure 2A**). Convolutional neural networks (CNNs) have gained widespread adoption in both regression and classification tasks over the past decade. This popularity is primarily due to their capability to autonomously learn deep, intricate features, a significant advancement over the traditional machine learning models that relied on manually extracted, handcrafted features (27). A fully connected neural network (FCNN) is a type of CNN that has fully connected hidden layers. A 1D FCNN model was previously implemented to correlate the mass density and RSP map based on DECT parametric maps (22). This 1D FCNN model was adapted to the SECT images dataset input in this study, including seven hidden layers (**Figure 2B**).

Due to the small size of the training set used in this study, overfitting issues could be associated with trained DL model outputs, which were also considered as vanishing gradient problems for our CNN models. A solution for the latter was proposed: ResNet (25). The ResNet implementation in this study includes a shortcut connection added between input and output after a few weight layers: the residual block. **Figure 2C** shows the ResNet workflow and **Figure 2D** shows the detail of the residual block in the ResNet. In **Figure 2C**, the input is linked to a convolutional layer, which is subsequently connected to four distinct sequences of residual blocks. Each type of residual block varies in terms of channel, kernel, stride, and padding. These details are illustrated in **Table 3**.

**Table 3:** Architecture of the 1D convolution components of ResNet (RN).

Network	Layer	Channel.	Kernel	Stride	Pad
	ConvA	64	7	2	3
Residual Block A1	Conv1_11	64	3	1	1
	Conv1_12	64	3	1	1
	Residual	64	1	2	0
Residual Block A2	Conv1_21	128	3	2	1
	Conv1_22	128	3	1	1
	Residual	128	1	2	0
Residual Block A3	Conv1_31	256	3	2	1
	Conv1_32	256	3	1	1
	Residual	256	1	2	0
Residual Block A4	Conv1_41	512	2	2	1
	Conv2_42	512	2	1	1
	Residual	512	1	2	0
Residual Block A5	Conv1_51	1024	3	2	1
	Conv2_52	1024	3	2	1
	Residual	1024	1	2	0

All the networks were trained using an NVIDIA GeForce 3090 GPU with 24 GB of memory, utilizing a batch size of 100. We set the learning rate for the Adam optimizer to 1e-5. During training, each batch optimization consumed 0.5 GB of CPU memory and 2 GB of GPU memory. All networks were implemented in PyTorch 2.1.

### 2.3 SECT stoichiometric method

A conventional physics-based SECT stoichiometric calibration method was utilized (2) to provide a standard set of reference values when comparing the DL models output for mass density and RSP. The X-ray linear attenuation coefficient µ of a material can be calculated using Equation (1):


(1) $$\mu =\rho \frac{N_{A}}{A}\left[ZK^{KN}\left(E\right)+Z^{n}K^{SCA}\left(E\right)+Z^{m}K^{PE}\left(E\right)\right]$$

where ρ is mass density, $N_{A}$ is Avogadro’s number, A is the atomic weight, Z is the atomic number, $K^{KN}$ , $K^{SCA}$ , and $K^{PE}$ are weighting constants for incoherent scattering, coherent scattering, and photoelectric effect, respectively, as a function of the SECT scanner X-ray spectrum (1). m and n are constants and were assigned 4.62 and 2.86 (28) for the energies encountered in kV X-ray imaging and the elements present in human tissue. Taking the ratio of the attenuation coefficient of material of interest to the attenuation coefficient of water, equation (2) can be derived:


(2) $$\frac{HU}{1000}+1={\hat{\rho }}_{e,w}\frac{\left[1+{\hat{Z}}^{1.86}k_{1}\left(E\right)+{\hat{Z}}^{3.62}k_{2}\left(E\right)\right]}{\left[1+{\hat{Z}}_{w}{}^{1.86}k_{1}\left(E\right)+{\hat{Z}}_{w}{}^{3.62}k_{2}\left(E\right)\right]}$$

where ${\hat{\rho }}_{e,w}$ is the electron density relative to water, ${\hat{Z}}_{w}$ is the effective atomic number of waters, $k_{1}=\frac{K^{SCA}}{K^{KN}}$ , $k_{2}=\frac{K^{PE}}{K^{KN}}$ . $k_{1}$ and $k_{2}$ were calculated by nonlinear regression, while the CT number values were taken as the mean value of each electron density insert from the SECT scan. Finally, the RSP of each material of interest was calculated using the Bethe equation (29):


(3) $$RSP={\hat{\rho }}_{e,w}\ln\left[\frac{2m_{e}c^{2}\beta^{2}}{I_{m}\left(1-\beta^{2}\right)}-\beta^{2}\right]/\ln\left[\frac{2m_{e}c^{2}\beta^{2}}{I_{water}\left(1-\beta^{2}\right)}-\beta^{2}\right]$$

where $m_{e}$ is the electron rest mass, c is the speed of light in vacuum, and β is the proton speed relative to the speed of light. $I_{m}$ and $I_{water}$ are the mean excitation energies of the material of interest and water. As recommended by ICRU49, $I_{water}$ was set to 75 eV (30). $I_{m}$ can be calculated from the atomic components using the Bragg additivity rule. Therefore, the stoichiometric calibration method can establish a functional relationship between CT numbers from the SECT datasets and RSP by Equation (2) and (3) (11).

### 2.4 Empirical model based on DECT parametric mapping

In addition to the stoichiometric calibration standard, a second set of reference values was obtained to compare the DL models output in terms of mass density and RSP prediction based on SECT images against the manufacturer-defined ground truth. These were based on an empirical model for mass density and RSP based on DECT parametric mapping (22), and corresponding definitions are shown in Equation (4) and (5) (7, 31). Therein, ρ denotes the mass density, and $\rho_{e}$ and $Z_{eff}$ are relative electron density and effective atomic number and were obtained from the DECT scanner console (Siemens Healthineers, syngo.via, Malvern, PA, USA). As used, the mass density model has a correction for the inflated lung. The RSP model includes corrections for different human tissues classified by various effective atomic numbers. The same phantoms were used in both DECT and SECT scans, and all tissue surrogate-defined contours were kept the same for reference comparison. Detailed information on DECT scans is listed in a previous publication (22).


(4) $$\rho =\{\begin{array}{c} -0.1746+1.176\rho_{e}\;\;\\ 0.26\;\;\;\;\;\;\;\;\;\;\;\;\; \end{array}\begin{array}{c} material\;\neq \;inflated\;lung\;\;\;\\ material\;=\;inflated\;lung\;\;\; \end{array}$$

(5) $$RSP=\{\begin{array}{c} \begin{array}{c} \rho_{e,}\;\;\;\;\;\;\;\;\;\;\;\;\;\;\;\;\;\;\;\;\;\;\\ \left(1.1114-0.0148Z_{eff}\right)\rho_{e}\\ 0.9905\rho_{e,}\;\;\;\;\;\;\;\;\;\;\;\;\;\;\;\; \end{array}\\ \left(1.1117-0.0116Z_{eff}\right)\rho_{e,} \end{array}\begin{array}{c} \;\;0\leq Z_{eff}<0.5\\ \begin{array}{c} 0.5\leq Z_{eff}<8.5\\ \begin{array}{c} 8.5\leq Z_{eff}<10\\ Z_{eff}\geq 10\;\;\;\;\;\;\;\; \end{array} \end{array} \end{array}$$

### 2.5 Evaluation metrics

The ground truth dataset included the reference mass density and RSP values as shown in **Table 1**. The CIRS M701 and M702 phantom images were manually contoured in RayStation 9B (RaySearch Laboratories, Stockholm, Sweden) for each of the tissue surrogate inserts, and reference values were assigned to each contour. Absolute percentage error (APE) and mean absolute percentage error (MAPE) were calculated as defined in Equation (6) and (7), where *i* denotes the *i^th^* voxel, x is the mass density or RSP at specific voxel, and N is the total number of voxels. The spatial distribution of error was also generated for the computed APE and displayed in error visualization maps of the whole phantom datasets.


(6) $$APE_{i}=\left|\frac{x_{i}-x_{i,REF}}{x_{i,REF}}\right|\times 100\%$$

(7) $$MAPE=\frac{1}{N}\sum_{i=1}^{N}APE_{i}$$

## 3 Results

### 3.1 Gammex electron density phantom analysis using pelvis protocol

**Table 4** presents results comparing MAPE of mass densities between DL models and the SECT stoichiometric method over seven Gammex electron density phantom tissue surrogates. As emphasized by the bold values, the DL models perform better on all tissue surrogates. For the lung tissue surrogates (LN300 & LN450), the DL models outperform conventional methods, while for higher density inserts, the FCNN and ResNet perform better than the traditional method in select cases. As seen in **Table 5**, a similar trend can be observed for the DL RSP predictions.

**Table 4:** Mass Density – MAPE [SECT DL vs. SECT stoichiometry].

Gammex Tissue Surrogates	LN450 Lung	Breast	Brain	Liver	B200 Bone Mineral	CB2-50%CaCO_3_ Bone	SB3 Cortical Bone
SECT Stoichiometric	12.57 ± 5.14	1.63 ± 1.36	2.23 ± 1.78	1.89 ± 1.03	1.18 ± 0.91	1.98 ± 1.12	2.32 ± 1.09
ANN	6.92 ± 4.81	1.95 ± 1.40	**1.34 ± 1.04**	**1.18 ± 0.90**	0.95 ± 0.74	6.22 ± 1.26	4.85 ± 1.28
FCNN	**6.43 ± 0.16**	1.18 ± 1.24	1.58 ± 1.18	1.69 ± 0.65	**0.45 ± 0.27**	1.36 ± 0.12	0.43 ± 0.30
ResNet	11.66 ± 0.27	**1.17 ± 1.37**	1.72 ± 1.11	1.66 ± 0.71	0.77 ± 0.23	**0.85 ± 0.38**	**0.29 ± 0.09**

Bold values mean best performance.

**Table 5:** RSP – MAPE [SECT DL vs. SECT stoichiometry].

Gammex Tissue Surrogates	LN450 Lung	Breast	Brain	Liver	B200 Bone Mineral	CB2-50%CaCO_3_ Bone	SB3 Cortical Bone
SECT Stoichiometric	3.71 ± 2.89	3.72 ± 2.09	4.50 ± 2.38	2.47 ± 1.67	1.64 ± 1.61	5.33 ± 1.10	7.99 ± 1.13
ANN	4.72 ± 3.54	3.33 ± 1.66	1.59 ± 1.12	**1.50 ± 0.92**	1.87 ± 0.73	4.81 ± 1.18	2.39 ± 1.10
FCNN	**2.18 ± 0.15**	**2.47 ± 1.47**	**1.16 ± 1.53**	1.78 ± 0.59	**1.39 ± 0.16**	**1.08 ± 0.11**	**1.96 ± 0.03**
ResNet	2.26 ± 0.24	2.55 ± 1.44	1.25 ± 1.46	1.73 ± 0.35	1.60 ± 0.18	1.66 ± 0.71	2.69 ± 0.14

Bold values mean best performance.

### 3.2 HN site analysis using HN protocol

The mass densities and RSP predictions from the empirical model based on DECT parametric mapping, the SECT stoichiometric method, and the DL model results analysis are shown in **Tables 6**, **7**. For all four tissues in M701, the DL methods are more accurate than the DECT empirical and SECT stoichiometric methods. The performance of FCNN and ResNet was comparable because of the similarity in the network fundamentals. For M702, the DECT empirical model outperforms the DL models in bone tissue prediction, which proves the advantage of DECT in bone tissue mass density estimation over SECT methods (ResNet has a comparable performance). However, this is not true for M701, the DL models based on SECT can achieve better results with the DECT empirical method in bone and other tissues. **Table 7** summarizes the RSP MAPE comparison between the DECT empirical model, the stoichiometric method, and the DL models. For M701, DL methods outperform the traditional methods, while for M702, the DECT empirical method outperforms the DL methods in the spinal cord.

**Table 6:** Mass Density – MAPE [SECT DL vs. SECT stoichiometry vs. DECT model].

M701	Brain	Spinal Cord	Soft Tissue	Bone
DECT empirical model	1.00 ± 0.80	1.40 ± 1.30	2.30 ± 2.20	2.50 ± 5.20
SECT Stoichiometric method	0.99 ± 0.66	1.53 ± 1.59	1.59 ± 1.72	4.79 ± 2.20
ANN	0.64 ± 0.50	0.96 ± 1.20	1.03 ± 1.08	1.95 ± 2.44
FCNN	0.39 ± 0.31	**0.59 ± 0.64**	**0.62 ± 0.52**	1.03 ± 1.30
ResNet	**0.15 ± 0.20**	0.74 ± 1.20	1.46 ± 1.03	**0.66 ± 0.85**
M702	Brain	Spinal Cord	Soft Tissue	Bone
DECT Empirical model	1.30 ± 0.08	1.20 ± 1.00	2.20 ± 1.50	**2.00 ± 1.90**
SECT Stoichiometric method	1.10 ± 0.97	1.80 ± 2.01	1.64 ± 2.12	6.80 ± 4.76
ANN	0.86 ± 0.81	1.28 ± 1.68	1.07 ± 1.70	4.84 ± 7.00
FCNN	0.89 ± 0.57	0.95 ± 1.20	**0.57 ± 1.15**	3.74 ± 6.78
ResNet	**0.19 ± 0.89**	**0.93 ± 2.18**	1.38 ± 2.44	2.16 ± 5.18

Bold values mean best performance.

**Table 7:** RSP – MAPE [SECT DL vs. SECT stoichiometry vs. DECT model].

M701	Brain	Spinal Cord	Soft Tissue	Bone
DECT Empirical model	1.20 ± 0.90	2.70 ± 1.90	2.90 ± 2.60	4.20 ± 4.90
SECT Stoichiometric method	0.95 ± 0.96	2.06 ± 1.53	2.96 ± 1.64	10.22 ± 3.00
ANN	1.15 ± 0.51	2.42 ± 0.85	1.11 ± 0.79	1.79 ± 1.68
FCNN	**0.85 ± 0.37**	**2.11 ± 0.64**	**1.08 ± 0.51**	0.67 ± 1.28
ResNet	1.85 ± 0.20	3.14 ± 0.83	2.24 ± 1.31	**0.48 ± 1.18**
M702	Brain	Spinal Cord	Soft Tissue	Bone
DECT Empirical model	0.90 ± 0.80	**1.60 ± 1.40**	2.60 ± 2.40	3.90 ± 2.20
SECT Stoichiometric method	1.24 ± 1.32	2.19 ± 1.84	3.20 ± 2.03	10.65 ± 4.47
ANN	0.99 ± 0.69	2.39 ± 1.44	1.03 ± 1.49	4.35 ± 5.57
FCNN	**0.45 ± 0.50**	1.80 ± 1.22	**0.74 ± 1.09**	3.00 ± 5.67
ResNet	1.82 ± 0.62	3.20 ± 1.56	2.18 ± 1.22	**1.67 ± 4.61**

Bold values mean best performance.

### 3.3 Chest site analysis using thorax protocol

**Table 8** summarizes the MAPE comparison of mass density estimated by empirical and DL models on the chest site using 120 kVp thorax protocol. For the lung site, the DECT empirical model doesn’t apply to the normal lung tissue (the empirical model assigns the inflated lung as constant mass density), so the lung tissue mass density prediction is not reported. **Table 9** shows the MAPE comparisons of RSP between the reference and DL models. All three DL models outperform the two reference models. The DECT empirical model and the stoichiometric method show better performance than ANN on lung tissue RSP prediction.

**Table 8:** Mass density – MAPE [SECT DL vs. SECT stoichiometry vs. DECT model].

M701	Spinal Cord	Soft Tissue	Bone	Lung
DECT Empirical model	3.40 ± 2.60	3.10 ± 2.50	3.40 ± 3.30	-^1^
SECT Stoichiometric method	1.53 ± 1.59	1.59 ± 1.72	3.68 ± 2.17	8.29 ± 7.34
ANN	0.96 ± 1.20	1.03 ± 1.08	3.64 ± 3.17	12.96 ± 7.09
FCNN	0.59 ± 0.64	**0.64 ± 1.13**	1.31 ± 2.36	**1.57 ± 0.39**
ResNet	**0.58 ± 0.61**	1.46 ± 1.56	**1.28 ± 1.43**	2.18 ± 0.73
M702	Spinal Cord	Soft Tissue	Bone	Lung
DECT Empirical model	2.50 ± 1.90	2.70 ± 2.20	4.00 ± 6.80	-^1^
SECT Stoichiometric method	1.72 ± 1.60	1.98 ± 4.10	5.77 ± 4.80	6.91 ± 6.35
ANN	1.32 ± 3.70	1.32 ± 3.70	4.55 ± 6.50	14.86 ± 7.37
FCNN	0.89 ± 0.71	**0.81 ± 3.65**	3.41 ± 6.54	**1.52 ± 0.49**
ResNet	**0.64 ± 0.83**	1.53 ± 3.87	**1.69 ± 4.87**	2.42 ± 1.97

^1^The DECT empirical model doesn’t apply to normal lung tissue.

Bold values mean best performance.

**Table 9:** RSP – MAPE [SECT DL vs. SECT stoichiometry vs. DECT model].

M701	Spinal Cord	Soft Tissue	Bone	Lung
DECT Empirical model	2.70 ± 2.60	3.20 ± 2.50	5.20 ± 3.60	7.00 ± 5.80
SECT Stoichiometric method	2.93 ± 2.35	3.06 ± 1.87	6.56 ± 2.94	8.29 ± 7.34
ANN	2.86 ± 1.36	1.07 ± 1.03	3.85 ± 2.88	12.36 ± 6.99
FCNN	**2.37 ± 0.95**	**1.04 ± 0.94**	1.73 ± 2.07	1.57 ± 0.43
ResNet	3.20 ± 0.75	2.14 ± 1.22	**0.72 ± 1.09**	**0.95 ± 0.72**
M702	Spinal Cord	Soft Tissue	Bone	Lung
DECT Empirical model	3.30 ± 3.10	2.70 ± 2.20	4.70 ± 6.20	6.70 ± 5.50
SECT Stoichiometric method	2.53 ± 1.95	3.49 ± 3.86	9.23 ± 4.25	6.75 ± 6.35
ANN	2.03 ± 1.56	1.32 ± 3.70	4.31 ± 5.60	14.23 ± 7.37
FCNN	**1.54 ± 1.30**	**0.89 ± 3.21**	2.94 ± 5.56	1.30 ± 0.54
ResNet	2.90 ± 1.64	2.27 ± 3.31	**1.36 ± 4.28**	**1.10 ± 0.70**

Bold values mean best performance.

### 3.4 Pelvis site analysis using pelvis protocol

**Table 10** shows the MAPE values of five models’ mass density predictions at the CIRS M701 and M702 phantom pelvis sites. DL methods show better performance in all three tissues and can reduce the error to<1% in the spinal cord and soft tissue. **Table 11** shows the MAPE values of three DL models and the two reference models’ RSP estimations at the CIRS M701 and M702 phantom pelvis sites. The DL methods have better performance in soft tissue and bone, and all models have comparable results in the spinal cord.

**Table 10:** Mass density – MAPE [SECT DL vs. SECT stoichiometry vs. DECT model].

M701	Spinal Cord	Soft Tissue	Bone
DECT Empirical model	3.30 ± 2.70	2.50 ± 2.00	2.40 ± 1.80
SECT Stoichiometric method	1.91 ± 1.62	2.07 ± 1.74	2.18 ± 2.00
ANN	1.05 ± 0.89	1.25 ± 1.03	3.15 ± 2.04
FCNN	0.73 ± 0.61	**0.75 ± 0.60**	**0.73 ± 1.12**
Resnet	**0.51 ± 0.43**	1.54 ± 1.05	1.19 ± 1.11
M702	Spinal Cord	Soft Tissue	Bone
DECT Empirical model	2.10 ± 1.80	2.30 ± 1.90	2.20 ± 1.70
SECT Stoichiometric method	1.82 ± 1.47	1.92 ± 1.74	3.76 ± 4.60
ANN	1.03 ± 0.88	1.20 ± 1.06	4.57 ± 5.09
FCNN	0.67 ± 0.57	**0.66 ± 0.54**	2.61 ± 5.62
Resnet	**0.53 ± 0.63**	1.41 ± 1.03	**1.79 ± 4.44**

Bold values mean best performance.

**Table 11:** RSP – MAPE [SECT DL vs. SECT stoichiometry vs. DECT model].

M701	Spinal Cord	Soft Tissue	Bone
DECT Empirical model	2.80 ± 2.20	2.60 ± 2.10	3.50 ± 2.30
SECT Stoichiometric method	**1.93 ± 1.62**	2.88 ± 1.64	6.56 ± 1.71
ANN	2.40 ± 1.45	1.25 ± 0.5	3.47 ± 1.91
FCNN	2.08 ± 1.18	**1.13 ± 0.64**	1.33 ± 1.12
Resnet	2.63 ± 1.31	2.22 ± 0.86	**0.61 ± 0.81**
M702	Spinal Cord	Soft Tissue	Bone
DECT Empirical model	2.50 ± 1.90	2.40 ± 1.90	3.40 ± 2.10
SECT Stoichiometric method	1.90 ± 1.23	3.06 ± 2.80	7.21 ± 3.67
ANN	2.20 ± 1.27	1.05 ± 0.85	4.69 ± 4.63
FCNN	**1.62 ± 0.92**	**0.74 ± 0.54**	2.75 ± 4.75
Resnet	2.70 ± 1.14	2.08 ± 0.86	**1.27 ± 3.77**

Bold values mean best performance.

### 3.5 Whole phantom site analysis using HN, thorax, and pelvis protocols

**Table 12** shows the MAPE comparisons of mass densities between DL models and the SECT stoichiometric method over the entire phantom site. **Table 13** summarizes the MAPE comparisons of RSP between DL models and the SECT stoichiometric method over the entire phantom site.

**Table 12:** Mass density – MAPE [SECT DL vs. SECT stoichiometry vs. DECT model].

M701	Brain	Spinal Cord	Soft Tissue	Bone	Lung
DECT Empirical model	1.00 ± 0.80	2.60 ± 2.40	2.70 ± 2.30	3.00 ± 3.80	-^1^
SECT Stoichiometric method	0.99 ± 0.66	2.34 ± 2.14	1.87 ± 1.86	3.17 ± 2.26	8.29 ± 7.34
ANN	0.64 ± 0.52	1.46 ± 1.64	1.29 ± 1.19	2.90 ± 2.36	12.96 ± 7.09
FCNN	0.39 ± 0.31	**0.92 ± 0.88**	**0.68 ± 0.86**	**0.92 ± 1.65**	**1.57 ± 0.39**
ResNet	**0.13 ± 0.20**	0.97 ± 1.54	1.42 ± 1.29	1.04 ± 1.45	2.18 ± 0.73
M702	Brain	Spinal Cord	Soft Tissue	Bone	Lung
DECT Empirical model	1.30 ± 0.90	1.90 ± 1.70	2.40 ± 2.00	2.50 ± 3.70	-^1^
SECT Stoichiometric method	1.10 ± 0.97	2.15 ± 1.52	1.92 ± 2.79	5.29 ± 4.89	6.91 ± 6.35
ANN	0.86 ± 0.81	1.53 ± 1.86	1.23 ± 2.35	4.65 ± 5.73	14.86 ± 7.37
FCNN	0.89 ± 0.57	1.09 ± 1.27	**0.70 ± 2.16**	3.19 ± 6.24	**1.52 ± 0.49**
ResNet	**0.19 ± 0.89**	**1.08 ± 2.29**	1.30 ± 2.48	**1.87 ± 4.82**	2.42 ± 1.97

^1^The DECT empirical model doesn’t apply to normal lung tissue.

Bold values mean best performance.

**Table 13:** RSP – MAPE [SECT DL vs. SECT stoichiometry vs. DECT model].

M701	Brain	Spinal Cord	Soft Tissue	Bone	Lung
DECT Empirical model	1.20 ± 0.90	4.40 ± 3.50	2.09 ± 2.40	3.20 ± 3.70	-^1^
SECT Stoichiometric method	0.95 ± 0.96	2.61 ± 2.10	2.08 ± 1.90	7.74 ± 2.78	8.62 ± 7.43
ANN	1.15 ± 0.51	2.74 ± 1.22	1.21 ± 1.17	3.03 ± 2.07	12.52 ± 7.39
FCNN	**0.85 ± 0.37**	**2.32 ± 0.87**	**0.75 ± 0.88**	**1.22 ± 1.34**	1.25 ± 2.37
ResNet	1.85 ± 0.20	2.73 ± 0.83	1.57 ± 1.31	1.26 ± 1.02	**1.01 ± 2.45**
M702	Brain	Spinal Cord	Soft Tissue	Bone	Lung
DECT Empirical model	0.90 ± 0.80	3.60 ± 3.00	2.50 ± 2.10	2.70 ± 3.50	-^1^
SECT Stoichiometric method	1.24 ± 1.32	2.44 ± 2.05	2.13 ± 2.81	8.87 ± 3.76	6.75 ± 1.36
ANN	0.99 ± 0.69	2.28 ± 1.60	1.23 ± 2.32	4.47 ± 4.65	14.23 ± 7.28
FCNN	**0.45 ± 0.50**	**1.71 ± 1.30**	**0.72 ± 2.16**	2.88 ± 4.70	1.30 ± 0.54
ResNet	1.82 ± 0.62	2.07 ± 1.65	1.56 ± 2.40	**1.42 ± 3.73**	**1.10 ± 0.70**

^1^The DECT empirical model doesn’t apply to normal lung tissue.

Bold values mean best performance.

**Figure 3:**
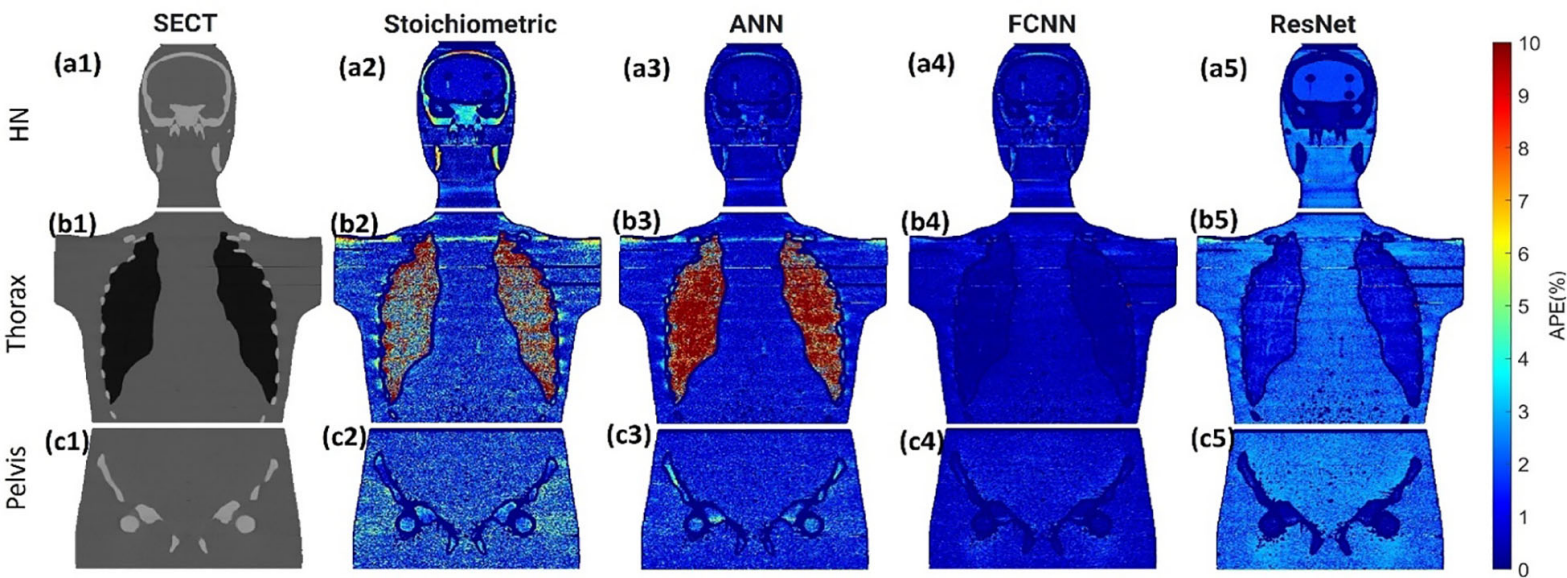
**(A1, B1, C1)** SECT images scan using different 120 kVp protocols. APE maps of mass densities between the reference and SECT parametric models at **(A2–A5)** HN, **(B2–B5)** thoracic, and **(C2–C5)** pelvic sites using CIRS M701 phantom.

**Figure 4:**
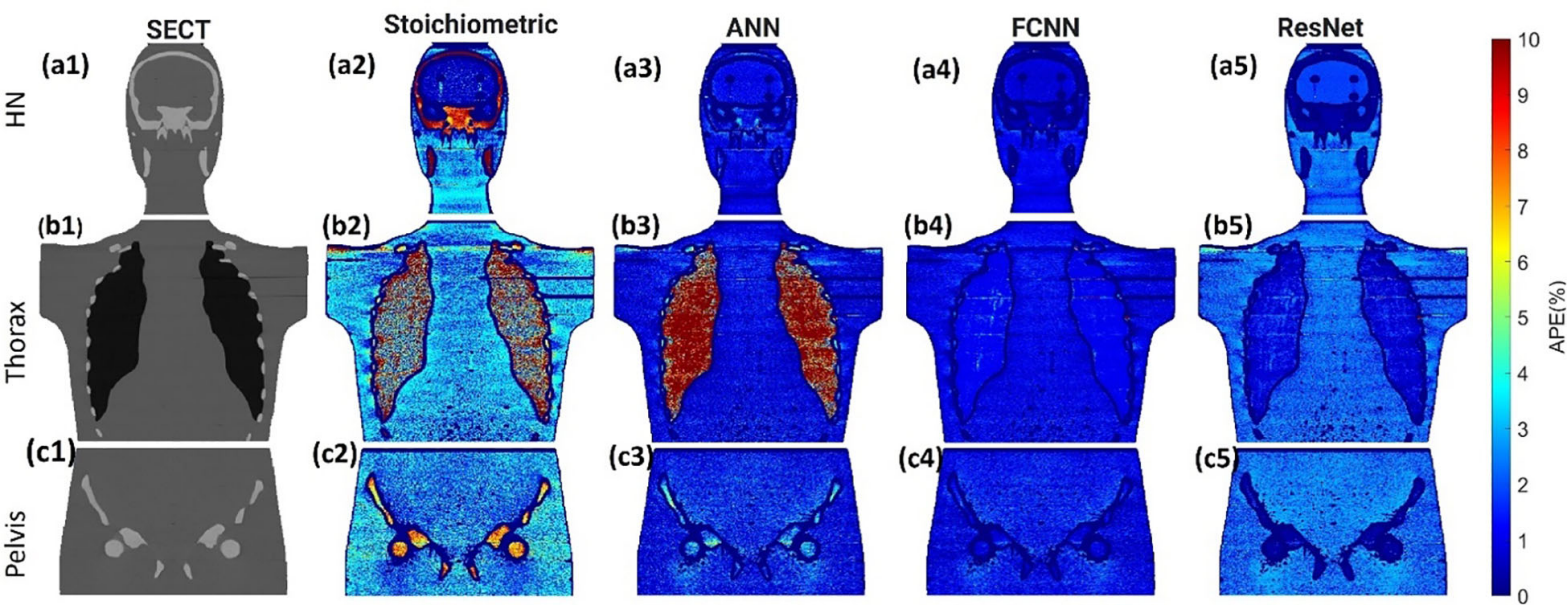
**(A1, B1–C1)** SECT images scan using different 120 kVp protocols. APE maps of RSP between the reference and SECT parametric models at **(A2–A5)** HN, **(B2–B5)** thoracic, and **(C2–C5)** pelvic sites using CIRS M701 phantom.

**Figure 3** illustrates the APE maps of the mass density estimation error. **Figures 3A17–C1** shows the SECT images of CIRS M701 phantom at three sites, HN, thorax, and pelvis, using 120 kVp corresponding scanning protocols. As presented using the APE color maps, the SECT stoichiometric model results in considerable uncertainty at lung and bone sites. Overall, the analysis for FCNN indicates the lowest error in the mass density estimation compared with other DL models. **Figure 4** illustrates the APE maps for RSP error estimation. As with mass density, the SECT stoichiometric method shows considerable error in RSP estimation, especially in bone and lung tissue. ANN improves the estimation accuracy for bone and soft tissue, while FCNN predictions indicate improvements overall. ResNet shows smaller APE for bone, specifically for the pelvis scan, even when compared with FCNN.

## 4 Discussions

External beam radiotherapy requires an accurate CT characterization of the patient geometry and heterogeneities to deliver accurate therapeutic patient doses. SECT imaging is the current clinical paradigm for generating the necessary information for clinical diagnosis and treatment planning in radiotherapy, including protons. Su et al. demonstrated the capability of machine learning methods to improve the prediction accuracy for mass density and RSP based on DECT imaging (18). In order to approach this methodology while using the more commonly available SECT imaging, we proposed a framework that can accurately predict mass density and RSP parametric maps for proton dose calculation while using SECT imaging as input. All SECT DL methods for mass density and RSP estimation were compared against the ground truth values defined in **Table 1** and compared to current standards for mass density and RSP parametrization (DECT and stoichiometry). All parametrization was done on phantom data since the DL network training requires accurate ground truth definition, which excludes patient data. The DECT model MAPE was consistently larger than that of the SECT stoichiometric method for some of the tissues (specifically for the spinal cord). This could be attributed to the fact that the DECT empirical model depends on the relative electron density for mass density prediction and effective atomic number for RSP, and it has not been calibrated for the specific CT scanner used in this work. The SECT DL models demonstrated good or better performance for parametrization based on three different clinical scanning protocols (HN, Chest, Pelvic). Note that DL models were trained with phantom data, while the SECT stoichiometric method and DECT empirical data were optimized for clinical use with both phantom and human tissue, which might lead to worse performance on anthropomorphic phantom mass density and RSP estimation.

Gomà et al. concluded that the Gammex phantom utilized in this study contains tissue substitutes better representative of the human body than the CIRS electron density phantom (17). As shown in **Tables 4**, **5**, we tested our DL models with Gammex 467 electron density phantom tissue surrogates, and the results indicate that the DL models can improve the mass density and RSP estimation relative to the stoichiometric method. Note that we tested our DL models on Gammex 467-1009 electron density phantom to evaluate the performance using different materials; then, we tested our DL models with CIRS M701&M702 phantoms for different materials and patient body sizes.

As shown in **Figures 3**, **4**, the SECT stoichiometric method yields larger error in the lung, skull, pelvic bone, and soft tissue than DL networks. Also, the mass density and RSP maps generated with the SECT stoichiometric method have more noise than those generated by DL models, which indicates that DL models can handle noise and artifact suppression superiorly. It was reported that the uncertainty associated with mass density estimation dominates the proton range calculation uncertainty (7, 15, 32). In this work, DL models were trained with SECT images of electron density phantom, and **Table 12** shows that the mass density MAPE can be improved significantly relative to the SECT stoichiometric method. The FCNN and ResNet outperform the ANN and reference models, and their MAPE values are close. A possible reason is that they share the same core network feature, the convolutional layer. Considering the training cost, the FCNN is recommended as the DL model for future dose calculation study. The RSP uncertainty is considerably larger than that of mass density, as demonstrated in **Table 13**, the DL models still exhibit the potential to enhance the accuracy of RSP estimation. FCNN could reduce the MAPE down to less than 2%, except in the spinal cord of CIRS M701.

**Figure 5:**
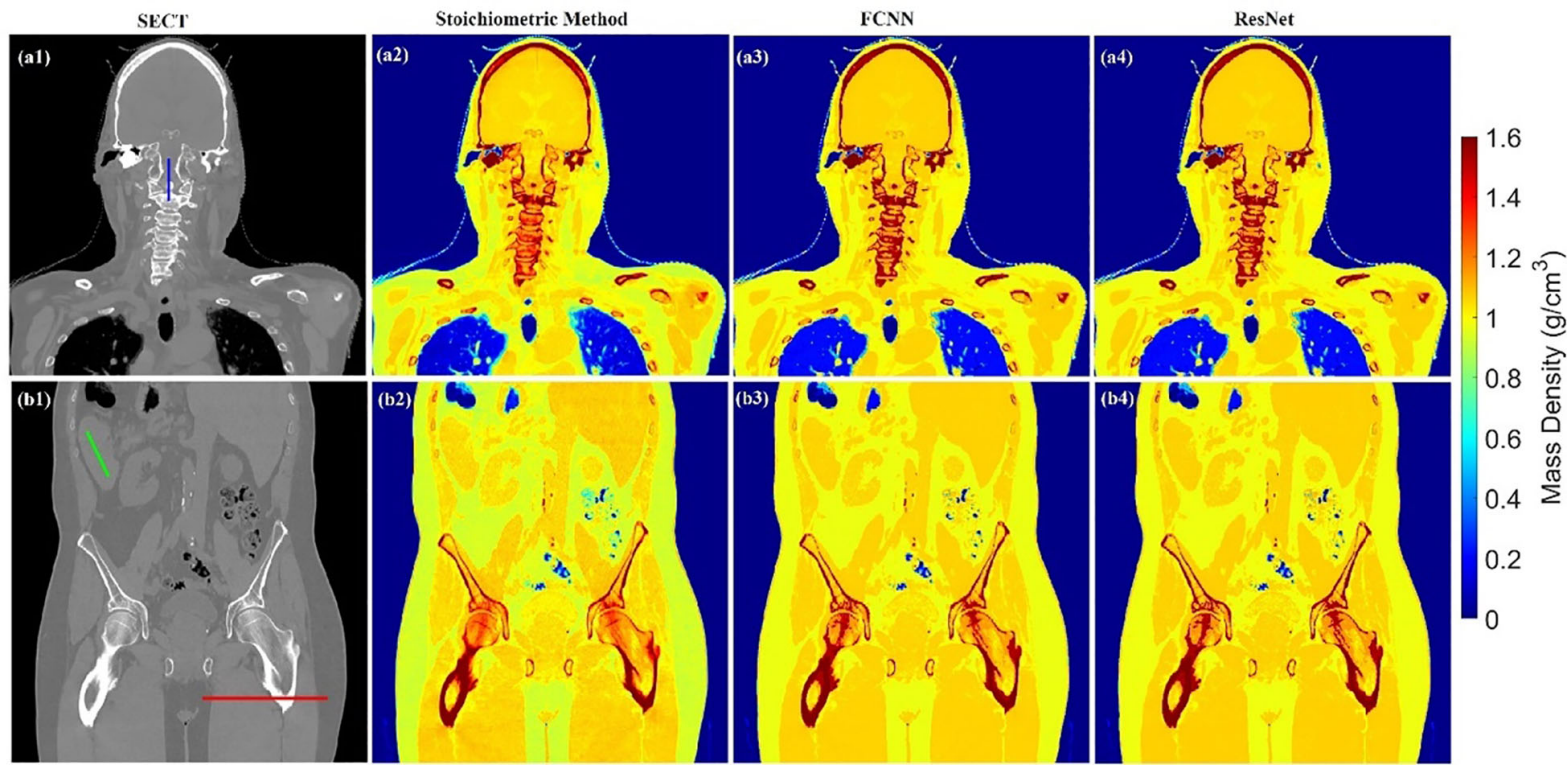
Mass density maps of two patients generated by different SECT FCNN and ResNet models at **A1–A4** and **B1–B4** using HN and pelvis scan.

**Figure 6:**
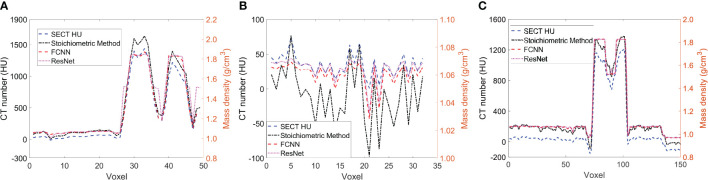
**(A)** the line profile from the blue line in **Figure 5A1**, **(B)** the line profile from green line in **Figure 5B1**, and **(C)** the line profile from the red line in **Figure 5B1**.

**Figure 5** illustrates the mass density prediction map generated by FCNN, ResNet, and the SECT stoichiometric method for two patients using HN and pelvis scans. As shown in **Figures 5A3, A4, B3, B4**, the DL models can reflect the patient’s anatomy qualitatively, compared with the SECT images in **Figure 5A1**. **Figures 6A–C** shows the comparison of CT number profile and density profile at the position marked in **Figures 5A1, B1**. **Figures 6A–C** demonstrates that the mass density profile generated by FCNN and ResNet has a strong agreement with the SECT number profile trend. As shown in **Figures 5A2–A4, B2–B4**, the mass density maps predicted by DL models have less noise than that predicted by the stoichiometric method, especially in the scan of the soft tissue of the pelvis. This implies that the DL models can suppress CT noises and artifacts, and it is also shown in the mass density profile comparison in **Figure 6** that the DL models’ mass density lines are smoother than that of the stoichiometric method. The empirical model can provide better contrast information between adipose and bone tissue than the DL models; this might be because the DL models predict a higher density (~0.96 g/cm^3^) for adipose compared to the stoichiometric method (~0.88 g/cm^3^). The accurate density of the patient’s adipose tissue is not known; DL models estimated it at 8% higher than the stoichiometric method because the training dataset of the DL models doesn’t cover the mass density range from 0.5 to 0.95 g/cm^3^, while the clinically used SECT stoichiometric method CT curve has been calibrated with over 30 materials on various mass densities. If more tissue surrogates can be adapted into the training set of this framework, the robustness of DL models will be significantly improved. As shown in **Figures 6A, C**, FCNN has better performance in reproducing minor structure information from SECT images, such as the mass density difference in pelvic bone and bone marrow. Because ResNet has a complex model structure, including deep CNN layers, for example, when the dimension of data is insufficient (only SECT image as input), the complex model does not necessarily lead to a robust result (33).

In supervised machine learning, a prevalent challenge is that DL models do not consistently generalize effectively from the training input data to new, unseen data (34). This overfitting phenomenon allows DL models to perform exemplarily in controlled conditions (similar or identical to the training conditions) while performing poorly on the application set. Many reasons can lead to this phenomenon, a lack of training dataset diversity being the predominant one. Only SECT phantom images were adapted into the training set; therefore, the DL networks’ infrastructure needed to be designed carefully to avoid overfitting. As shown in **Figures 5**, **6**, the mass density profile estimated by DL models can basically follow the trend of SECT CT number and overlap with the profile of the SECT stoichiometric method. As shown in **Table 14**, DL models predicted a patient tissue mass density similar to that predicted using traditional methods. Integrating these findings, the design of the DL network successfully addresses and mitigates the overfitting issue.

**Table 14:** Average mass density of three tissues comparison among DL models and the Stoichiometric model based on one patient SECT scan.

Patient	Brain (g/cm^3^)	Bone (g/cm^3^)	Lung (g/cm^3^)
SECT Stoichiometric method	1.04 ± 0.04	1.69 ± 0.18	0.20 ± 0.05
ANN	1.04 ± 0.02	1.62 ± 0.17	0.17 ± 0.04
FCNN	1.05 ± 0.01	1.66 ± 0.17	0.20 ± 0.01
ResNet	1.05 ± 0.03	1.68 ± 0.15	0.21 ± 0.01

## 5 Conclusion

A DL framework was proposed to improve the mass density and RSP parametrization for proton dose calculation based on the SECT images. All three DL models outperform the SECT stoichiometric method in tissue substitute except the lung surrogate. FCNN and ResNet improved the mass density and RSP estimation accuracy based on SECT images, outperforming the SECT stoichiometric method over the entire phantom, and outperforming the DECT empirical model. DL models also demonstrate the ability to suppress CT image noise. The proposed DL frameworks have the potential to improve the clinical proton dose calculation based on SECT scans.

## Data availability statement

The data analyzed in this study is subject to the following licenses/restrictions: The dataset can be available by contact the corresponding author. Requests to access these datasets should be directed to xyang43@emory.edu.

## Ethics statement

The studies involving humans were approved by Emory University review board (IRB#114349). The studies were conducted in accordance with the local legislation and institutional requirements. The ethics committee/institutional review board waived the requirement of written informed consent for participation from the participants or the participants’ legal guardians/next of kin because this is a retrospective study, and the patient’s information was anonymized.

## Author contributions

YG: Data curation, Investigation, Methodology, Writing – original draft, Writing – review & editing. C-WC: Investigation, Software, Validation, Writing – review & editing. JR: Writing – review & editing. MA: Writing – review & editing. YL: Methodology, Software, Writing – review & editing. SP: Methodology, Writing – review & editing. JB: Supervision, Writing – review & editing. JZ: Investigation, Methodology, Writing – review & editing. TL: Writing – review & editing. XY: Conceptualization, Funding acquisition, Investigation, Supervision, Writing – review & editing.

## References

[1] Zhu J Penfold SN. Dosimetric comparison of stopping power calibration with dual-energy CT and single-energy CT in proton therapy treatment planning. Med Phys (2016) 43:2845–54. doi: 10.1118/1.4948683 27277033

[2] Schneider U Pedroni E Lomax A. The calibration of CT Hounsfield units for radiotherapy treatment planning. Phys Med Biol (1996) 41:111–24. doi: 10.1088/0031-9155/41/1/009 8685250

[3] Woodard HQ White DR. The composition of body tissues. Br J Radiol (1986) 59:1209–18. doi: 10.1259/0007-1285-59-708-1209 3801800

[4] International Commission On Radiation, U. & Measurements B. M. D. Tissue substitutes in radiation dosimetry and measurement. (Bethesda, MD (United States): International Commission on Radiation Units and Measurements) (1989). Available at: https://www.osti.gov/biblio/10102048.

[5] Scott JA. Photon, electron, proton and neutron interaction data for body tissues ICRU report 46. International commission on radiation units and measurements, Bethesda 1992, $40.00. J Nucl Med (1993) 34:171–1.

[6] McCollough CH Boedeker K Cody D Duan X Flohr T Halliburton SS. Principles and applications of multienergy CT: Report of AAPM Task Group 291. Med Phys (2020) 47:4. doi: 10.1002/mp.14157 32215937

[7] Hunemohr N Krauss B Tremmel C Ackermann B Jakel O Greilich S. Experimental verification of ion stopping power prediction from dual energy CT data in tissue surrogates. Phys Med Biol (2014) 59:83–96. doi: 10.1088/0031-9155/59/1/83 24334601

[8] Paganetti H. Range uncertainties in proton therapy and the role of Monte Carlo simulations. Phys Med Biol (2012) 57:R99–117. doi: 10.1088/0031-9155/57/11/R99 22571913 PMC3374500

[9] Paganetti H Jiang H Parodi K Slopsema R Engelsman M. Clinical implementation of full Monte Carlo dose calculation in proton beam therapy. Phys Med Biol (2008) 53:4825–53. doi: 10.1088/0031-9155/53/17/023 18701772

[10] Taasti VT Bäumer C Dahlgren CV Deisher AJ Ellerbrock M Free J. Inter-centre variability of CT-based stopping-power prediction in particle therapy: Survey-based evaluation. Phys Imaging Radiat Oncol (2018) 6:25–30. doi: 10.1016/j.phro.2018.04.006 33458385 PMC7807627

[11] Chang CW Huang S Harms J Zhou J Zhang R Dhabaan A. A standardized commissioning framework of Monte Carlo dose calculation algorithms for proton pencil beam scanning treatment planning systems. Med Phys (2020) 47:1545–57. doi: 10.1002/mp.14021 31945191

[12] Peters N Wohlfahrt P Dahlgren CV De Marzi L Ellerbrock M Fracchiolla F. Experimental assessment of inter-centre variation in stopping-power and range prediction in particle therapy. Radiother Oncol (2021) 163:7–13. doi: 10.1016/j.radonc.2021.07.019 34329653

[13] Paganetti H. Monte Carlo simulations will change the way we treat patients with proton beams today. Br J Radiol (2014) 87:20140293. doi: 10.1259/bjr.20140293 24896200 PMC4112394

[14] Bourque AE Carrier J-F Bouchard H. A stoichiometric calibration method for dual energy computed tomography. Phys Med Biol (2014) 59:2059–88. doi: 10.1088/0031-9155/59/8/2059 24694786

[15] Yang M Virshup G Clayton J Zhu XR Mohan R Dong L. Theoretical variance analysis of single- and dual-energy computed tomography methods for calculating proton stopping power ratios of biological tissues. Phys Med Biol (2010) 55:1343–62. doi: 10.1088/0031-9155/55/5/006 20145291

[16] Wohlfahrt P Mohler C Hietschold V Menkel S Greilich S Krause M. Clinical implementation of dual-energy CT for proton treatment planning on pseudo-monoenergetic CT scans. Int J Radiat Oncol Biol Phys (2017) 97:427–34. doi: 10.1016/j.ijrobp.2016.10.022 28068248

[17] Gomà C Almeida IP Verhaegen F. Revisiting the single-energy CT calibration for proton therapy treatment planning: a critical look at the stoichiometric method. Phys Med Biol (2018) 63:235011. doi: 10.1088/1361-6560/aaede5 30474618

[18] Su KH Kuo JW Jordan DW Van Hedent S Klahr P Wei Z. Machine learning-based dual-energy CT parametric mapping. Phys Med Biol (2018) 63:125001. doi: 10.1088/1361-6560/aac711 29787382

[19] Schmidhuber J. Deep learning in neural networks: An overview. Neural Networks (2015) 61:85–117. doi: 10.1016/j.neunet.2014.09.003 25462637

[20] Sahiner B Pezeshk A Hadjiiski LM Wang X Drukker K Cha KH. Deep learning in medical imaging and radiation therapy. Med Phys (2019) 46:e1–e36. doi: 10.1002/mp.13264 30367497 PMC9560030

[21] Lecun Y Bengio Y Hinton G. Deep learning. Nature (2015) 521:436–44. doi: 10.1038/nature14539 26017442

[22] Chang C-W Gao Y Wang T Lei Y Wang Q Pan S. Dual-energy CT based mass density and relative stopping power estimation for proton therapy using physics-informed deep learning. Phys Med Biol (2022) 67(11):115010. doi: 10.1088/1361-6560/ac6ebc PMC10410526 35545078

[23] Hassoun MH. Fundamentals of artificial neural networks. Cambridge, Massachusetts, United States: MIT press (1995).

[24] Krizhevsky A Sutskever I Hinton GE. Imagenet classification with deep convolutional neural networks. Adv Neural Inf Process Syst (2012) 25.

[25] He K Zhang X Ren S Sun J. Identity mappings in deep residual networks. In Computer Vision–ECCV 2016: 14th European Conference, Amsterdam, The Netherlands, October 11–14, 2016, Proceedings, Part IV 14. (Springer International Publishing) (2016), 630–45.

[26] Paszke A Gross S Massa F Lerer A Bradbury J Chanan G. Pytorch: An imperative style, high-performance deep learning library. Adv Neural Inf Process Syst (2019) 32.

[27] Kiranyaz S Avci O Abdeljaber O Ince T Gabbouj M Inman DJ. 1D convolutional neural networks and applications: A survey. Mechanical Syst Signal Process (2021) 151:107398. doi: 10.1016/j.ymssp.2020.107398

[28] Rutherford RA Pullan BR Isherwood I. Measurement of effective atomic number and electron density using an EMI scanner. Neuroradiology (1976) 11:15–21. doi: 10.1007/BF00327253 934468

[29] Bichsel H. Passage of charged particles through matter. California Univ., Berkeley (USA), Dept. of Physics: Office of Scientific and Technical Information (OSTI) (1969).

[30] Deasy J. ICRU report 49, stopping powers and ranges for protons and alph particles. Med Phys (1994) 21:709–10. doi: 10.1118/1.597176

[31] Beaulieu L Tedgren Å.C Carrier J-F Davis SD Mourtada F Rivard MJ. Report of the Task Group 186 on model-based dose calculation methods in brachytherapy beyond the TG-43 formalism: Current status and recommendations for clinical implementation. Med Phys (2012) 39:6208–36. doi: 10.1118/1.4747264 23039658

[32] Yang M Zhu XR Park PC Titt U Mohan R Virshup G. Comprehensive analysis of proton range uncertainties related to patient stopping-power-ratio estimation using the stoichiometric calibration. Phys Med Biol (2012) 57:4095–115. doi: 10.1088/0031-9155/57/13/4095 PMC3396587 22678123

[33] Chang C-W Dinh NT. Classification of machine learning frameworks for data-driven thermal fluid models. Int J Thermal Sci (2019) 135:559–79. doi: 10.1016/j.ijthermalsci.2018.09.002

[34] Ying X. An overview of overfitting and its solutions. J Physics: Conf Ser (2019) 1168:022022. doi: 10.1088/1742-6596/1168/2/022022

